# Effect of Clear Corneal Incisions via Femtosecond Laser Versus Manual Incisions on Corneal Aberrations in Cataract Surgery

**DOI:** 10.3390/mi16080939

**Published:** 2025-08-15

**Authors:** Vesko Onov, Gabriele Thumann, Martina Kropp, Zeljka Cvejic, Filip Slezak, Bojan Pajic

**Affiliations:** 1 Eye Clinic ORASIS, Swiss Eye Research Foundation, 5734 Reinach, Switzerland; onov.corporation@gmail.com (V.O.); filip.slezak@orasis.ch (F.S.); 2 Division of Ophthalmology, Department of Clinical Neurosciences, Geneva University Hospitals, 1205 Geneva, Switzerland; gabriele.thumann@hug.ch (G.T.); martina.kropp@unige.ch (M.K.); 3 Experimental Ophthalmology, University of Geneva, 1205 Geneva, Switzerland; 4 Department of Physics, Faculty of Sciences, University of Novi Sad, 21000 Novi Sad, Serbia; zeljka.cvejic@df.uns.ac.rs; 5 Faculty of Medicine of the Military Medical Academy, University of Defense, 11000 Belgrade, Serbia

**Keywords:** HOAs = higher-order aberrations, surgically induced astigmatism, femtosecond laser (fs), femtosecond laser-assisted cataract surgery (FLACS)

## Abstract

This study aimed to evaluate whether clear corneal incisions (CCIs) created with the FEMTO LDV Z8 femtosecond laser during cataract surgery are non-inferior to manual CCIs in terms of surgically induced astigmatism (SIA) and higher-order aberrations (HOAs). A total of 78 cataract patients were randomly assigned to two groups: 38 eyes underwent femtosecond laser-assisted cataract surgery (FLACS), and 40 eyes underwent conventional manual cataract surgery (CCS). Preoperative and six-week postoperative SIA, HOAs, and all topographic and refractive data were analysed for both groups. FLACS-generated CCIs demonstrated equivalence to manual CCIs. The mean SIA was 0.44 ± 0.27 dioptres (D) in the FLACS group and 0.58 ± 0.46 D in the CCS group (*p* = 0.18), with lower variability in the FLACS group. The root mean square (RMS) corneal HOA at six weeks was 0.69 ± 0.17 µm in the FLACS group and 0.80 ± 0.56 µm in the CCS group (*p* > 0.05). These results confirm the efficacy, reproducibility, and safety of FLACS. Although not statistically significant, FLACS induced numerically lower SIA values and less variability than manual CCIs. Both groups were comparable in terms of HOAs, though higher mean values and variability were observed in the CCS group.

## 1. Introduction

With the advent of modern technologies, ophthalmological procedures of all kinds have become more precise and easier to perform. Femtosecond lasers have been used in corneal surgery for over 16 years and remain the most common and modern instrument for correcting vision through refractive surgery [1,2,3]. In the context of cataract surgery, the laser first assists in fragmenting the cataract lens by applying laser pulses in a predetermined pattern, then it precisely creates a near-perfect circular anterior capsulotomy with a predetermined diameter, and finally it can create a small incision in the peripheral cornea for lens removal and replacement [4,5,6]. Femtosecond laser cataract surgery usually involves a capsulotomy and lens fragmentation, but the incisions are often made manually with a scalpel. The wavelength of the femtosecond laser is in the near-infrared spectrum, which is not absorbed by optically clear tissue at low power densities [5]. Due to the design of the laser, an incision can be made within the ocular tissue without having to make an incision that does not follow the study protocol or is on the outside of the structure or, in other words, the inside of a cataract lens without damaging the surface of the eye or cornea.

As far as incisions are concerned, the femtosecond laser allows the surgeon to choose the position of the incision as well as its exact size and architecture [7]. Femtosecond laser CCIs are highly precise, reproducible, and stable. They are self-sealing and have excellent wound geometry and are potentially subject to less mechanical trauma during the procedure [8]. SIAs have been reported to be smaller with femtosecond laser CCIs than with manual CCIs for both temporal and superior oblique incisions with other systems, but the difference was not statistically significant [8].

The introduction of low-energy femtosecond laser technology represents a significant advancement in cataract surgery. Unlike high-pulse energy lasers, which rely on mechanical disruption, low-energy systems enable precise tissue separation with minimal collateral damage and reduced mechanical tearing [9,10,11]. Improvements in the numerical aperture of the laser focusing optics and the repetition rate of the laser sources have further reduced collateral damage while increasing precision. Increasing the numerical aperture of the focusing optics reduces the pulse energy threshold for optical breakthrough, resulting in cutting with virtually no collateral damage [11].

With regard to incisions, FLACS allows for individual planning and precise execution of incisions in terms of position, exact size, and architecture based on intraoperative OCT [7]. Femtosecond laser CCIs have proven to be highly precise, individually customisable, reproducible, and stable [12]. Several studies have shown that, due to their uniform and consistent cutting morphology, FS lasers provide better self-sealing and thus more resistant incisions than conventional cataract surgery [12,13,14,15]. However, even when using FLACS, many surgeons still perform incisions and side-port incisions manually.

The aim of this study was to assess whether CCIs created using the FEMTO LDV Z8 femtosecond laser in FLACS are non-inferior to manually created CCIs in conventional cataract surgery (CCS) regarding surgically induced astigmatism (SIA) and higher-order aberrations (HOAs).

## 2. Materials and Methods

This prospective, randomised clinical trial was conducted at the Orasis Eye Clinic in Reinach, Switzerland (NCT04082273), in accordance with the tenets of the Declaration of Helsinki. Ethical approval was obtained from the Ethics Committee of the Kantonal Ethikkomission Nordwest- und Zentralschweiz in Basel, Switzerland (BASEC-ID: 2019-01166), and all patients signed informed consent forms before surgery. Where both eyes were operated on, the same method was used, but only one eye was used for this study. Randomisation consisted of deciding which surgical method would be used and which eye would be selected and used for the evaluation. From August 2019 to January 2024, patients were eligible if they were aged 40 years or older, diagnosed with cataracts suitable for phacoemulsification with primary intraocular lens (IOL) implantation, able to cooperate with the femtosecond laser docking system, and willing to attend all follow-up visits. Exclusion criteria included glaucoma, pseudoexfoliation syndrome, small pupils, a history of corneal surgery, other ophthalmological diseases, corneal scarring, or pterygium. None of the patients had undergone eye surgery prior to this study, nor did any of the patients have intraoperative floppy iris syndrome (IFIS). All patients underwent a comprehensive preoperative ophthalmic evaluation. Cataract severity was graded using the Lens Opacities Classification System III (LOCS III), based on nuclear opalescence (grades 0 to 5) [16]. Patients were randomly assigned (1:1) to undergo either femtosecond laser-assisted cataract surgery (FLACS) or conventional cataract surgery. When both eyes of a subject were eligible for inclusion, the subject was randomly assigned to undergo surgery on either the left or right eye.

The primary objective was to determine whether CCIs created with the FEMTO LDV Z8 laser were non-inferior to manual CCIs in CCS, as assessed by surgically induced astigmatism (SIA) six weeks postoperatively. A mean SIA value for eyes in the FLACS group corresponding to the SIA value for eyes in the CCS group at a range of 0.30 dioptres was considered positive evidence for the equivalence of the FEMTO LDV Z8 (Ziemer Ophthalmic Systems AG, Port, Switzerland) CCIs to the manual CCIs. Secondary objectives included the evaluation of the difference in the efficacy between FLACS with the FEMTO LDV Z8 laser and CCS with respect to corneal higher-order aberrations (HOAs) (6 weeks postoperatively), intraoperative evaluation of the precision of FEMTO LDV Z8 OCT automatic eye structure detection, and the evaluation of the safety of FEMTO CCIs compared to manual CCIs in terms of the rate of intra- and postoperative CCI-related complications. Corneal topography and aberration measurements were obtained using the Galilei G2 system (Ziemer Ophthalmic Systems AG, Port, Switzerland).

### 2.1. Surgical Technique

All cataract surgeries were performed by a single experienced surgeon (B.P.). Preoperative pupil dilation was achieved using Mydriasert (phenylephrine hydrochloride 5.4 mg and tropicamide 0.28 mg) (Théa Laboratories, Clermont-Ferrand, France) inserted into the inferior fornix 45 min prior to surgery. Intracameral anaesthesia (lidocaine hydrochloride and epinephrine, 0.005 mg) was administered. Regardless of the surgical method, the main incision was performed horizontally in the right eye and at 12 h in the left eye.

In the FLACS group, a femtosecond laser (Ziemer LDV Z8) (Ziemer Ophthalmic Systems AG, Port, Switzerland) was used to create a 2.2 mm clear corneal incision, a capsulorhexis, and to pre-fragment the nucleus. Laser parameters followed standard manufacturer guidelines [17,18,19,20].

During FLACS, the eye is stabilised using a suction ring applying a target vacuum of 400 mbar. Prior to positioning the laser handpiece onto the suction ring, balanced salt solution (BSS) is applied to the liquid interface to ensure proper optical coupling. The femtosecond laser cutting sequence starts with an anterior capsulotomy targeting a 5 mm diameter. Subsequently, lens fragmentation is performed following a spider web pattern—dividing the lens into six radial segments intersected by a concentric circle. Clear corneal incisions are then created to access the anterior chamber. The primary incision is consistently planned at 2.2 mm, accompanied by two additional side-port incisions measuring 0.8 mm each. The shape of the main incision was biplanar, and each side-port incision was uniplanar.

**Figure 1:**
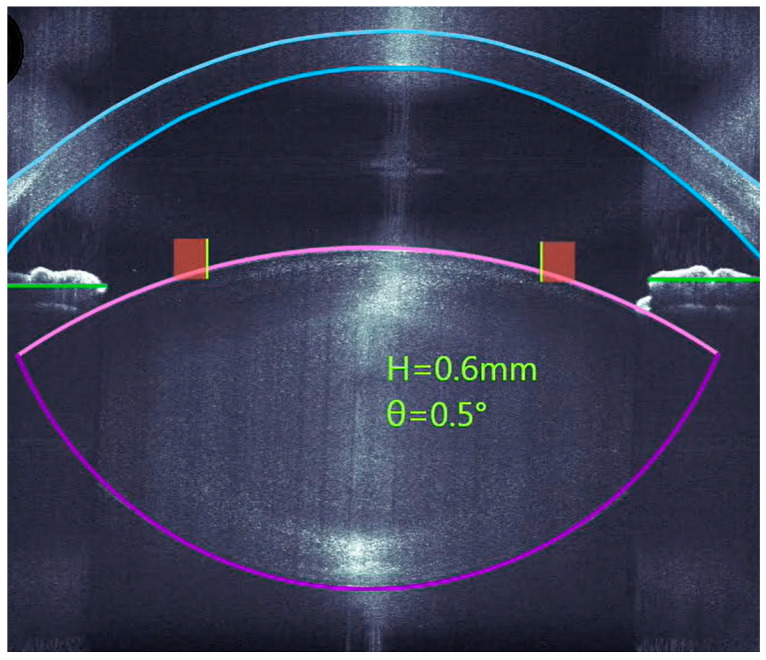
An intraoperative OCT cross-section for planning the capsulotomy and lens fragmentation.

With the intraoperative OCT application, the site of application can be defined very precisely. The planned diameter of the capsulotomy and the thickness of the lens can be seen very precisely in the cross-section of the OCT images (Figure 1), where it can be seen exactly how deep the incisions of the lens fragmentation should be.

**Figure 2:**
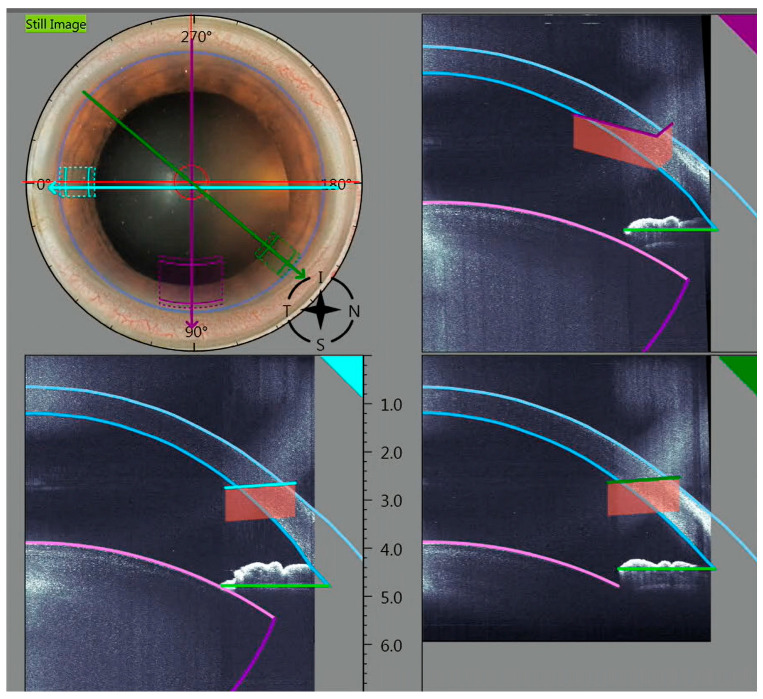
An intraoperative image with the corresponding OCT images for determining the main incision to the side-ports.

Figure 2 shows the planned main incision and side-port generated by an image with simultaneous OCT and how the incisions must be positioned exactly.

After laser application, hydration is carefully performed between the lens and lens capsule until the nucleus becomes mobile for phacoemulsification. A Catharex 3 (Oertli Instruments AG, Berneck, Switzerland) was used for phacoemulsification in both groups. The pre-fragmented lens pieces were then aspirated using a high vacuum of up to 600 mmHg at a flow of 50 mL/min, and phacoemulsification was performed. All patients received a monofocal aspheric IOL (KOWA 2.2R) (Kowa Company, Ltd., Tokyo, Japan) implanted into the capsular bag.

In the group where conventional cataract surgery (CCS) was performed, two uniplanar side-port incisions were carried out using a lance with a 0.8 mm diameter. An ophthalmic viscoelastic device (OVD) was applied to the anterior chamber. A capsulorhexis was applied using a cystotome with a target diameter of 5 mm. The main incision of 2.2 mm was then created. The main incision has a biplanar shape. Hydrodissection mobilises the lens nucleus until it rotates. The hydrodissection was performed through the main incision so that the vitreous body is not hydrated. Phacoemulsification was performed using the Phaco-Chop technique. The phaco parameters applied were the same as those in the FLACS group. After performing I/A with the removal of the cortex, the folded IOL was implanted into the capsular bag using a standard preloaded injector instrument.

### 2.2. Statistical Analysis

SPSS version 22 was used to statistically analyse the demographic data described and the clinical measurements used in this study. The statistical power calculation was performed before the start of this prospective study. The calculation confirms that the sample size of 78 patients with 78 eyes provides 80% power for α = 0.05 to detect moderate effect sizes (Cohen’s d ≈ 0.5). All SIA calculations were performed using vectorial calculation according to the method described by Alpins [21,22]. Depending on the data distribution, parametric or non-parametric tests were used. *p*-values below 0.05 were considered statistically significant. The descriptive statistics of the continuous variables were calculated, including the mean, standard deviation, median, and minimum and maximum values. The distributions of the continuous variables were analysed to assess the normality of the sample, with normality confirmed by the Shapiro and Kolmogorov–Smirnov test. When non-parametric tests were required, the Wilcoxon rank sum test and the Mann–Whitney U test were used for non-normally distributed data. For normally distributed data, the parametric paired *t*-test was used. A one-way analysis of variance was performed to compare the mean values of the different measurements during the surgical sessions. The Pearson correlation coefficient was used to assess the correlation between different variables. The statistical significance of the correlation was tested using the stratified data on cataract grading, anterior chamber depth, and corneal radii.

## 3. Results

A total of 78 patients (45 men, 33 women) were enrolled: 38 underwent femtosecond laser-assisted cataract surgery (FLACS), and 40 underwent conventional cataract surgery (CCS). The mean age of the total cohort was 69.4 ± 10.4 years (FLACS: 69.5 ± 10.2; CCS: 69.4 ± 10.6), with no statistically significant difference between the two groups (0.941). Unilateral cataract surgery was performed on the right eye in 37 patients and on the left eye in 41 patients (19 right eyes and 19 left eyes in the FLACS group and 18 right eyes and 22 left eyes in the CCS group). The mean cataract grade based on the LOCS III scale after LOCS III was significantly higher when comparing the FLACS group (3.13 ± 0.81) to the CCS group (2.33 ± 0.68; *p* < 0.05). Randomisation was determined before the start of this study. The demographic distribution is shown in Table 1.

### 3.1. Surgically Induced Astigmatism After 6 Weeks

**Figure 3:**
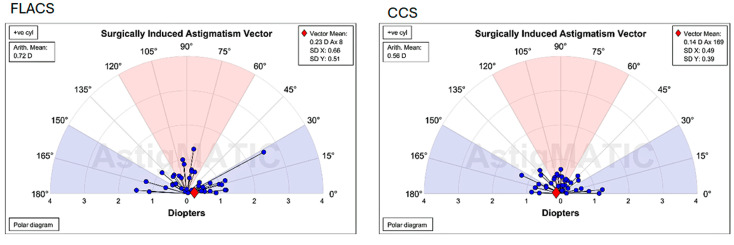
Angle plots via Alpin’s method for surgically induced astigmatism (SIA) in both groups.

The primary aim of this study, to demonstrate that CCIs created with the FEMTO LDV Z8 laser are equivalent to manually created CCIs in conventional cataract surgery in terms of SIA 6 weeks after cataract surgery, was achieved. The mean ± standard deviation (SD) of surgically induced astigmatism (SIA) was 0.35 ± 0.33 dioptres (D) in the FLACS group and 0.45 ± 0.37 D in the CCS group. Although the difference in SIA between the groups did not reach statistical significance (*p* = 0.18), the CCS group exhibited mean SIA values and greater variability compared to the FLACS group, as shown in the Alpins vector plot (Figure 3). Notably, the calculated effect size was moderate (Cohen’s d = 0.37; 95% CI: 0.02–0.72), suggesting a potentially meaningful clinical difference that may not be fully captured by *p*-values alone. The polar diagrams illustrate the vectors of surgically induced astigmatism (SIA) computed with Alpins’ approach. Each blue dot signifies the astigmatic variation in magnitude and axis for an individual eye. The red diamond signifies the centroid (mean vector) of SIA for the group. This graphical form enables the comparison of astigmatic outcomes by illustrating both the direction and dispersion of SIA vectors among patients.

The full vectorial parameters for all eyes in both the FLACS and CCS groups, including target-induced astigmatism (TIA), difference vector (DV), and correction index (CI), are shown in Supplementary Table S1.

### 3.2. Higher-Order Aberrations (HOAs)

**Figure 4:**
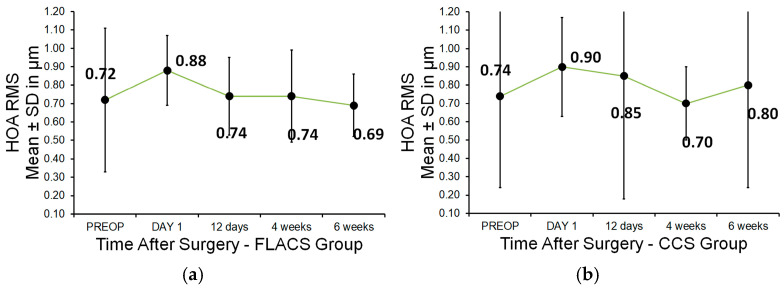
(**a**,**b**) Distribution of total corneal HOAs preoperatively and 1 day, 12 days, 4 weeks, and 6 weeks postoperatively. (**a**) FLACS group. (**b**) CCS group.

No statistically significant differences in corneal higher-order aberrations (HOAs) were observed between the FLACS and CCS groups at any time point: preoperatively or postoperatively on day 1, day 12, week 4, or week 6. The mean (±SD) HOA values for the FLACS and CCS groups were the following: preoperative: 0.72 ± 0.39 vs. 0.74 ± 0.5; day 1: 0.88 ± 0.19 vs. 0.9 ± 0.27; day 12: 0.74 ± 0.21 vs. 0.85 ± 0.67; week 4: 0.74 ± 0.25 vs. 0.7 ± 0.2; week 6: 0.69 ± 0.17 vs. 0.8 ± 0.56 (*p* > 0.05 for all visits) (Figure 4). Although not statistically significant, the HOAs in the CCS group showed a higher mean and standard deviation six weeks after surgery compared to both the preoperative value and the FLACS group. However, a statistically significant difference in horizontal coma was detected between groups at six weeks postoperatively (*p* = 0.049; paired *t*-test), with the FLACS group showing lower values. The mean (±SD) horizontal coma value six weeks postoperatively was −0.15 (±0.28) µm in the FLACS group and 0.0 (±0.33) µm in the CCS group. There was no statistically significant difference in vertical coma between the two groups.

The Pearson correlation was used to analyse the correlation between HOAs and the preoperative anterior chamber depth and corneal radii. No significant correlation was found for either anterior chamber depth or corneal radii in relation to HOAs (*p* > 0.05). There was also no significant correlation between HOAs and which eye, right or left, was operated on (*p* > 0.05). In this study, the total corneal HOAs were measured and analysed.

As the FLACS group had a statistically significantly higher mean cataract grade than the CCS group, an additional stratified analysis was performed to assess the impact of cataract grade on HOAs. Patients were categorised into three cataract grade subgroups: Grade ≤ 2; Grade = 3; Grade ≥ 4. Among patients with LOCS III Grade ≤ 2, the FLACS group exhibited significantly lower HOA values compared to the CCS group (*p* = 0.002). In all other LOCS III grade subgroups, the difference was not statistically significant (*p* > 0.05).

### 3.3. Endothelial Cell Density (ECD)

In the FLACS group, the mean corneal endothelial cell count (CD) was 2428 ± 353 cells/mm^2^ preoperatively, 2323 ± 380 cells/mm^2^ 1 day postoperatively, 2322 ± 384 cells/mm^2^ 12 days postoperatively, 2295 ± 387 cells/mm^2^ 4 weeks postoperatively, and 2213 ± 397 cells/mm^2^ 6 weeks postoperatively. In the CCS group, the mean preoperative corneal endothelial cell count (CD) was 2434 ± 324 cells/mm^2^, 2326 ± 380 cells/mm^2^ 1 day postoperatively, 2311 ± 400 cells/mm^2^ 12 days postoperatively, 2261 ± 417 cells/mm^2^ 4 weeks postoperatively, and 2254 ± 417 cells/mm^2^ 6 weeks postoperatively.

There was no statistically significant difference between the groups preoperatively (*p* = 0.856), 1 day postoperatively (*p* = 0.776), 12 days postoperatively (*p* = 0.280), 4 weeks postoperatively (*p* = 0.432), and 6 weeks postoperatively (*p* = 0.225). The multivariate regression, especially the ANOVA, was not significant for either the FLACS (*p* = 0.142) or the CCS (*p* = 0.989).

### 3.4. Central Corneal Thickness (CCT)

There was no statistically significant difference between the FLACS and CCS groups at any of the measurement time points (1 day, 12 days, 4 weeks, and 6 weeks). The CCT values (mean ± SD in µm) for the FLACS and CCS groups were as follows: preoperative: 573.9 ± 37.1 vs. 574.7 ± 45.6; after 1 day: 586.0 ± 69.1 vs. 576.4 ± 77.7; after 12 days: 589.1 ± 42.8 vs. 582.1 ± 42.0; after 4 weeks: 577.7 ± 38.2 vs. 575.9 ± 41.9; after 6 weeks: 572.6 ± 37.3 vs. 571.9 ± 41.3.

No intraoperative or postoperative complications related to CCIs were observed in either group throughout the six-week follow-up period.

## 4. Discussion

The Ziemer LDV Z8 femtosecond laser used in this study operates at high frequency and low pulse energy, features which have been associated with minimised collateral tissue damage and enhanced wound architecture. These properties may contribute to reduced surgically induced astigmatism and postoperative high-order aberrations, supporting the observed clinical outcomes.

Previous studies have demonstrated that high-pulse-energy femtosecond lasers can induce greater mechanical and thermal stress on corneal tissue, potentially resulting in microcavitation bubbles, irregular stromal surfaces, and elevated postoperative higher-order aberrations (HOAs). In contrast, the Ziemer LDV Z8 system used in our study operates at low pulse energy and high frequency, allowing for smoother lamellar cuts and reduced collateral tissue effects. This laser–tissue interaction profile may explain the relatively low postoperative HOAs observed in the treated eyes. These findings are in line with recent comparative reports on flap quality and aberration induced across femtosecond platforms, reinforcing the advantage of low-energy systems for optical outcomes in cataract and refractive surgery [23,24].

This study compared surgically induced astigmatism (SIA) and higher-order aberrations (HOAs) following cataract surgery using a 2.2 mm main incision and two 0.8 mm side-port incisions in both femtosecond laser-assisted (FLACS) and conventional (CCS) surgical groups, from the preoperative period to six weeks postoperatively. The results demonstrated that femtosecond laser-assisted incisions are comparable to manual incisions in terms of SIA and HOA outcomes. The SIA values calculated using Alpins vector analysis showed a numerically lower mean and reduced variability in the FLACS group compared to the CCS group. This suggests that FLACS may offer improved reproducibility and standardisation compared to CCS, which in turn could lead to more predictable refractive outcomes. Theoretically, SIA and HOAs might be expected to be higher in the FLACS group due to the more central positioning of clear corneal incisions compared to manual limbal incisions. With regard to SIA and HOAs, the sections in our study are on par with those in CCS and FLACS, which has also been established by other comparable studies [25,26,27,28]. An analysis of individual HOA components revealed significantly lower horizontal coma values in the FLACS group compared to CCS, consistent with the findings in previous studies [28].

Our results are consistent with most other studies that have also found less variability and lower numerical SIA values for FLACS [8,13,25,29,30]. A retrospective study compared FLACS to the high-energy Catalys platform with manual cataract surgery in 104 eyes and showed a significantly greater SIA value in the FLACS cohort at 1 week (*p* = 0.02) and 1 month (*p* = 0.04) but no significant difference in SIA at 3 months (*p* = 0.11) [30]. Some studies using alternative high-energy femtosecond platforms (e.g., Catalys, Victus) reported conflicting results regarding SIA. One study noted significantly greater SIA values in the FLACS group at 1 week and 1 month postoperatively [29], whereas another reported significantly lower SIA values at 6 months with the FLACS group (0.35 D vs. 0.9 D; *p* = 0.015) [30]. However, a direct comparison of studies is only possible to a limited extent, as different FS laser platforms, different incision sizes (>2.2 mm incisions), different architectures of the manual incisions, different follow-up durations, and different methods were used to analyse SIA. Evidence suggests that smaller incisions (2.0–2.2 mm) are associated with lower SIA values compared to larger incisions (>2.75 mm) [31,32,33]. Additionally, temporal incisions generally induce less astigmatism than superiorly positioned ones [34]. In the present study, 2.2 mm incisions were performed in the superior position according to the surgeon’s established standard technique. Interestingly, no significant differences in SIA or HOAs were found between the left and right eyes, despite differing main incision placements (superiorly at 12 o’clock for left eyes and horizontally for right eyes).

Both the anterior and posterior corneal surfaces are known to influence overall corneal astigmatism and thus surgically induced astigmatism (SIA). In the past, SIA was assessed solely by anterior corneal measurements; however, recent research has emphasised the significant influence of the posterior cornea, particularly in eyes with minimal astigmatism. Recent evidence has shown that overlooking posterior corneal astigmatism can lead to the inaccurate assessment of overall corneal astigmatism and surgically induced astigmatism (SIA) [35,36,37]. The posterior cornea is particularly prone to causing astigmatism against the rule, which may compensate for the changes observed on the anterior surface after surgery against the rule. As a result, in the Discussion Section, we explained that the heterogeneity observed in the SIA results may be due in part to unmeasured changes in posterior corneal curvature that are not reflected in conventional keratometry or anterior topography measurements. Furthermore, our results show that corneal HOAs did not differ significantly between the two groups over the entire follow-up period. At six weeks, the CCS group exhibited a higher mean and greater variability in HOA values compared to both their preoperative values and the FLACS group, although the difference was not statistically significant. Corneal HOAs are known to reduce retinal image quality and can impair visual performance, particularly contrast sensitivity, night vision, and subjective quality of vision [38,39,40,41]. They can lead to symptoms such as difficulty seeing at night, glare, haloes, blurred vision, starbursts, and diplopia. Previous studies have also found no induced corneal aberrations after uniplanar, biplanar, or triplanar FS laser corneal incisions and no differences between manual and FS laser CCIs within the central 5.0 or 6.0 mm zone of the cornea [14,23,24]. Other studies show that FLACS induced significantly fewer higher-order aberrations (HOAs) than conventional phacoemulsification, and especially in combination with multifocal or enhanced-depth-of-focus IOLs, FLACS was associated with improved image quality and higher patient satisfaction [41,42].

No intraoperative or postoperative complications were observed in our study. The considerably higher mean cataract grades (LOCS III) observed in the FLACS group may be interpreted as a limitation, given their potential impact on phaco time, phaco energy, and HOAs. Our findings indicate that FLACS using the FEMTO LDV Z8 does not induce additional mechanical or thermal trauma to the cornea, despite the higher cataract grades in the FLACS group. This is consistent with published results [9,10,41,43] and confirms that femtosecond laser-assisted cataract surgery (FLACS) with the FEMTO LDV Z8 platform has promising safety results and offers precise surgical performance without an increased risk of postoperative complications.

Recent studies provide important comparative insights into refractive and pachymetric stability after FS-LASIK, PRK, and SMILE, supporting the notion that femtosecond laser techniques can provide improved corneal regularity and biomechanical stability [44]. In addition, the reported molecular findings indicate different wound healing responses associated with different laser-based refractive procedures [45]. These findings usefully contribute to our interpretation of the potential benefits of FLACS in reducing corneal tissue destruction and a milder inflammatory response, factors that may be important in minimising postoperative corneal aberrations and improving long-term visual quality.

Although patient-reported outcomes (PROs) were not collected in this study, the reduction in higher-order aberrations (HOAs), particularly spherical aberration and coma, is of therapeutic importance. Numerous studies indicate that increased higher-order aberrations (HOAs) correlate with reduced contrast sensitivity, increased glare and haloes, and an overall deterioration in visual quality—elements that directly impact patient satisfaction in practice.

A correlation between postoperative HOAs and reduced contrast sensitivity and night vision disturbances has already been reported in previous studies [46]. Other studies have also supported this aspect, showing that patients with reduced higher-order aberrations (HOAs) had less dysphotopsia and better subjective visual quality after cataract surgery. Consequently, the observed reduction in higher-order aberrations in the FLACS cohort could significantly improve postoperative visual comfort and the overall quality of life [47].

## 5. Conclusions

The high-frequency, low-energy femtosecond laser LDV Z8 clear corneal incisions resulted in numerically lower SIA induction values with lower variability compared to manual CCIs, even though no statistical significance was achieved. Both groups were also comparable in terms of HOAs, with higher mean values and higher variability in the CCS group, despite the more central positioning of the laser incisions, prone to higher induction. The planned incision with the auto-detection system did not require adjustment in any case and supported efficient workflows.

## Figures and Tables

**Table 1:** Patient demographics.

	Total (*n* = 78)	FLACS (*n* = 38)	CCS (*n* = 40)
**Age (years)**			
Mean ± SD	69.5 ± 1.4	69.5 ± 10.2	69.4 ± 10.6
Median	71	70.5	71.5
(Min/Max)	(43/95)	(47/95)	(43/90)
**Gender (eyes)**			
Male, *n* (%)	45 (57.7)	20 (52.6)	25 (62.5)
**Eye**			
OS, *n* (%)	41 (52.6)	19 (50)	22 (55)
OD, *n* (%)	37 (47.4)	19 (50)	18 (45)
**LOCS III**			
Mean ± SD	2.72 ± 0.74	3.13 ± 0.81	2.33 ± 0.68
(Min/Max)	(1/5)	(2/5)	(1/4)
Grade 1, *n*	2	0	2
Grade 2, *n*	34	9	25
Grade 3, *n*	25	16	9
Grade 4, *n*	15	12	3
Grade 5, *n*	1	1	0

## Data Availability

Data supporting the findings of this study are available upon reasonable request from the first and corresponding authors. The datasets are archived at the clinics where the patients were treated. Data are not publicly available due to privacy concerns.

## Supplementary Materials

The following supporting information can be downloaded at: https://www.mdpi.com/article/10.3390/mi16080939/s1, Table S1: Vectorial SIA Parameters.

## Abbreviations

The following abbreviations are used in this manuscript:

CCIs	clear corneal incisions
SIA	induced corneal astigmatism
HOAs	higher-order aberrations
FLACS	femtosecond laser-assisted cataract surgery
CCS	conventional cataract surgery
OCT	optical coherence tomography
LOCSIII	Lens Opacities Classification System III
Yb:YAG	ytterbium-doped yttrium aluminium garnet crystal
SESAM	semiconductor saturable absorber mirror
BSS	balanced salt solution
IFIS	intraoperative floppy iris syndrome
