# Supplementary material for: Effect of Clear Corneal Incisions via Femtosecond Laser Versus Manual Incisions on Corneal Aberrations in Cataract Surgery
Source: Micromachines (Basel). 2025 Aug 15;16(8):939. doi: 10.3390/mi16080939 (PMC12388711; doi:10.3390/mi16080939)
Supplement: Supplementary file 1 [file micromachines-16-00939-s001.zip › micromachines-3777838-supplementary.pdf]

Supplementary Table S1: Vectorial SIA Parameters It includes: **TIA (Target-Induced Astigmatism):** preoperative astigmatism; **SIA (Surgically Induced Astigmatism)**: postoperative astigmatism; **DV (Difference Vector)**: vector difference between SIA and TIA; **CI (Correction Index):** ratio of SIA to TIA

FLACS Eyes	TIA (D)	SIA (D)	DV (D)	CI	CCS Eyes	TIA (D)	SIA (D)	DV (D)	CI
**1**	0.62	0.32	0.3	0.52	**1**	0.79	0.4	0.39	0.51
**2**	0.19	0.88	1.07	0.13	**2**	1.14	0.63	0.51	0.55
**3**	0.28	0.25	0.53	0.89	**3**	0.34	0.62	0.98	1.82
**3**	0.83	0.03	0.86	0.04	**3**	0.72	0.01	0.73	0.01
**5**	0.38	0.08	0.46	0.21	**5**	0.54	0.05	0.59	0.09
**6**	0.73	0.23	0.96	0.32	**6**	0.56	0.37	0.19	0.66
**7**	0.29	0.23	0.52	0.79	**7**	2.27	0.01	2.26	0
**8**	0.53	0.02	0.55	0.04	**8**	0.07	0.86	0.93	12.29
**9**	1.22	0.23	1.45	0.19	**9**	0.26	0.41	0.67	1.58
**10**	0.69	0.49	0.2	0.71	**10**	1.78	1.2	0.58	0.68
**11**	1.35	0.68	0.67	0.5	**11**	0.42	0.39	0.81	0.93
**12**	0.93	0.43	0.5	0.46	**12**	0.22	0.51	0.73	2.32
**13**	0.95	0.59	1.54	0.62	**13**	0.2	0.43	0.63	2.15
**14**	0.87	0.53	0.34	0.61	**14**	0.28	0.5	0.78	1.79
**15**	0.28	0.64	0.92	2.28	**15**	0.61	0.45	1.06	0.74
**16**	0.19	0.28	0.47	1.47	**16**	0.73	1.12	1.85	1.53
**17**	0.83	0.31	1.14	0.37	**17**	0.32	0.19	0.51	0.59
**18**	0.49	0.29	0.2	0.59	**18**	0.44	0.03	0.47	0.07
**19**	0.84	0.04	0.8	0.05	**19**	0.94	0.06	0.88	0.06
**20**	0.63	0.1	0.73	0.16	**20**	0.45	0.12	0.57	0.25
**21**	0.12	0.12	0.24	1	**21**	1.82	0.52	2.34	0.29
**22**	0.28	0.13	0.41	0.46	**22**	1.81	0.97	0.84	0.54
**23**	0.85	0.06	0.91	0.07	**23**	2.35	0.45	2.8	0.19
**24**	0.39	0.3	0.09	0.77	**24**	1	0.02	1.02	0.02
**25**	2.13	0.15	2.28	0.07	**25**	0.67	0.37	1.04	0.55
**26**	0.87	0.54	0.33	0.62	**26**	0.5	0.86	1.36	1.72
**27**	0.6	0.07	0.67	0.12	**27**	0.46	0.24	0.22	0.52
**28**	0.24	0.52	0.76	2.17	**28**	1.09	1.77	1.86	1.62
**29**	1.94	0.23	1.71	0.12	**29**	3.54	0.44	3.98	0.12
**30**	1.14	0.58	0.56	0.51	**30**	0.9	0.54	1.44	0.6
**31**	0.33	0.19	0.52	0.58	**31**	1.18	0.22	1.3	0.19
**32**	1.2	1.82	3.02	1.52	**32**	1.44	0.14	1.58	0.1
**33**	1.6	0.57	1.03	0.36	**33**	0.44	0.2	0.64	0.45
**34**	0.98	0.44	1.42	0.45	**34**	1.12	0.2	0.92	0.18
**35**	1.23	0.35	1.58	0.31	**35**	1.16	0.78	0.48	0.67
**36**	2.12	0.15	2.27	0.07	**36**	1.88	0.63	1.25	0.34
**37**	0.77	0.26	1.03	0.34	**37**	0.64	0.18	0.82	0.28
**38**	1.54	0.04	1.58	0.03	**38**	2.22	0.43	1.79	0.19
					**39**	0.55	0.02	0.57	0.04
					**40**	3.23	0.7	3.93	0.22
